# The learning curve for minimally invasive Oxford phase 3 unicompartmental knee arthroplasty: cumulative summation test for learning curve (LC-CUSUM)

**DOI:** 10.1186/s13018-014-0081-8

**Published:** 2014-09-06

**Authors:** Qidong Zhang, Qian Zhang, Wanshou Guo, Zhaohui Liu, Liming Cheng, Debo Yue, Nianfei Zhang

**Affiliations:** Department of joint surgery, China-Japan Friendship Hospital, Yinghua Street, Beijing, 100029 People’s Republic of China; Beijing University of Chinese Medicine, Yinghua Street, Beijing, 100029 People’s Republic of China

**Keywords:** Unicompartmental knee arthroplasty, Total knee arthroplasty, Minimally invasive surgery, Learning curve, CUSUM analysis

## Abstract

**Background:**

The minimally invasive Oxford unicompartmental knee arthroplasty (UKA) is a demanding procedure but has many advantages compared with total knee arthroplasty (TKA). The aim of this observational study was to investigate the learning curve of one experienced surgeon introducing minimally invasive Oxford phase 3 UKA into his routine clinical practice.

**Methods:**

The first 50 consecutive cases of minimally invasive Oxford phase 3 UKA performed by one surgeon were evaluated to determine whether there was an association between outcomes and the cumulative number of cases performed, indicating the presence of learning curve. The cohort was divided into two groups: group A comprised the first 25 cases and group B cases 26–50. Duration of surgery, blood loss, Hospital for Special Surgery score, range of motion, complications, and the radiographical position of the implant were compared between the groups. The cumulative summation test for learning curve (LC-CUSUM) was then used to further analyze the learning curve.

**Results:**

The mean age and follow-up were 64.4 years and 50.9 months, respectively. The duration of surgery and blood loss were significantly more favorable in group B. The length of incision gradually reduced from 9.7 ± 1.3 to 8.5 ± 1.1 cm. Failures were identified in nine patients (18%). Two revisions and two dislocations were encountered in group A; one revision was performed 4 years after surgery for a patient in group B because of a fracture. One case of lateral compartment osteoarthritis was identified in group A. Two patients in each group reported continuing unexplained pains. CUSUM analysis showed that failure rates diminished rapidly after 16 cases and reached an acceptable rate after 29 cases.

**Conclusions:**

Minimally invasive Oxford phase 3 UKA for anteromedial osteoarthritis is a demanding procedure, but satisfactory outcomes can be achieved after approximately 25 cases.

## Background

As the proportion of elderly people in the global population rises, the incidence of knee osteoarthritis is increasing. Unicompartmental knee arthroplasty (UKA) is a treatment option for patients with osteoarthritis of the medial compartment. As surgical techniques and instruments have improved, this procedure has shown many advantages over more traditional techniques, such as less soft tissue injury, a smaller incision, minimal bone resection, preservation of normal knee kinematics, reduced hospital stay, and more rapid recovery [1-4]. In addition, it could be argued that it is reasonable to undertake a UKA if osteoarthritis is only present in one compartment. The mobile Oxford medial UKA (Oxford® Unicompartmental Knee, Biomet, Bridgend, UK) has been used successfully for more than three decades to treat anteromedial arthritis of the knee. The phase 3 implant was introduced in 1998 and has been widely adopted, with many reports of excellent outcomes [5-7].

A learning curve is defined as an improvement in performance over time or with increasing experience or training. The starting point of the curve in surgery indicates the baseline level of surgical skill, and the end point shows an assumed expertise level. The slope of the curve indicates the speed of learning, which may be influenced by surgeon-related factors and institutional factors, such as operating team experience, the size of the institution, caseload volume, and financial resources [8,9]. Most surgeons performing joint replacements had a great deal of experience in total knee arthroplasty (TKA) before UKA procedure; however, the surgical principles, techniques, and management of UKA and TKA are different and UKA can be very challenging. Good clinical practice and the introduction of new orthopedic techniques mandate the need to determine the nature of the learning curve. The cumulative summation method (CUSUM) is a sequential analysis tool that was initially used in industrial settings for quality control purposes. It can be used to establish the learning curve for a surgical procedure and allows one to judge when an individual’s performance has achieved a predefined level of competence. The aim of this study was to establish the learning curve of an experienced knee surgeon introducing minimally invasive Oxford phase 3 UKA into routine clinical practice using the CUSUM technique.

## Methods

Approval of the study was obtained from the institutional review board. From January 2009 to March 2010, 50 consecutive UKA cases performed by senior author were included in the analysis, with no loss to follow-up.

The indication for UKA was anteromedial osteoarthritis with severe medial knee pain and radiographic evidence of osteoarthritis in the medial compartment. Other indications were an intact anterior cruciate ligament (ACL), varus deformity <15°, flexion contracture <15°, and an intact lateral compartment [10]. The preoperative diagnosis was primary anteromedial osteoarthritis in all patients.

All patients were placed in the supine position on a standard operating table after spinal anesthesia had been administered. A tourniquet was applied to the proximal thigh on the operative side and inflated to 300 mm Hg. A medial parapatellar incision was used, and the patella was not everted. Non-anatomic bearings were used in all cases.

Clinical outcomes were evaluated by measuring the difference between the range of knee motion (ROM) and Hospital for Special Surgery (HSS) knee score before surgery and at final follow-up. Weight-bearing anteroposterior and lateral radiographs of the knee were obtained, as well as a long hip-to-ankle film to assess the femorotibial angle and implant position. Loosening of the tibial or femoral components was identified by an area of radiolucency >2 mm around the components. Overrotation of the component was diagnosed if the alignment angle exceeded 10°. Each evaluation was made twice by two independent observers. The end point for survival was defined as revision for any reason. Patient-related information was collected using a standardized questionnaire administered before surgery and at follow-up.

### Statistical analysis

All data were analyzed using SPSS version 17.0 (SPSS Inc., Chicago, IL, USA). Data are reported as the mean with the standard deviation. The chi-squared test and Student’s *t* test were used to determine whether there were statistically significant differences between the groups. A *p* value < 0.05 was considered statistically significant [11].

### CUSUM analysis

For CUSUM analysis, four parameters are defined: the acceptable failure rate (*p*0), the unacceptable failure rate (*p*1), the type I error rate (*α*), and the type II error rate (*β*). The equations shown in Table 1 are used to calculate the CUSUM score [12]. On the basis of a literature review, we defined the acceptable UKA failure rate as 20% and the unacceptable failure rate as 40% [2,13]. The probabilities of *α* and *β* were set at 0.05 and 0.20, respectively. Failure situations were defined as revision, reoperation, postoperative complications, and radiographic malposition. The results of CUSUM analysis are presented in a chart with case numbers plotted on the *x*-axis and the corresponding CUSUM score on the *y*-axis, which allows performance over consecutive procedures to be visualized. When a failure occurred, the constant ‘1 − *S*’ was added to the cumulative score. When a success occurred, the variable ‘*s*’ was subtracted from the cumulative score. Hence, success is rewarded by a downward slope whereas failure is represented by an upward slope on the chart. If the line crosses the upper decision limit (h1) from below, this indicates that the actual failure rate is equal to the unacceptable failure rate with a type I error. If the line crosses the lower decision limit (h0) from above, this indicates that the actual failure rate does not differ from the acceptable failure rate with a type II error probability of 0.20. When the line is between h1 and h0, no statistical inference can be made.

**Table 1:** **CUSUM equations and variables**

**Variable**	**Value**
*p*0—acceptable UKA failure rate	0.20
*p*1—unacceptable UKA failure rate	0.40
*α*—probability of the type I error	0.05
*β*—probability of the type II error	0.20
*P* = ln (*p*1/*p*0)	0.69
*Q* = ln [(1 − *p*0)/(1 − *p*1)]	0.29
*s* = *Q*/(*P* + *Q*)	0.29
1 − *S*	0.71
*a* = ln [(1 − *β*) / *α*]	2.77
*b* = ln [(1 − *α*) / β]	1.56
*h*0 = −*b* / (*P* + *Q*)	−1.59
*h*1 = *a* / (*P* + *Q*)	2.83

## Results

One patient in group A died from lung cancer 34 months after surgery, but there were no symptoms or clinical signs of implant failure or radiographic signs of loosening at the last follow-up. The mean follow-up was 50.9 ± 4.9 months (range 45–60 months) after the final assessment in December 2013. The mean age on the day of surgery was 64.4 ± 8.5 years. Twenty-seven UKAs were performed on the right knee and 23 on the left; 16 patients were male and 34 female. There were no significant differences in any of the clinical characteristics of the patients in each group.

The mean duration of surgery decreased with the number of cumulative cases, from 85.0 ± 15.7 min in group A to 64.4 ± 13.9 min in group B (*p* < 0.05). Perioperative blood loss was also lower in group B (185.0 ± 69.2 ml compared with 226.2 ± 74.8 ml in group A; *p* < 0.05). The mean incision length was 9.7 ± 1.3 cm in group A compared with 8.5 ± 1.1 cm in group B (*p* = 0.001). In all patients, passive full flexion of the knee and painless active full flexion was possible within 7 postoperative days and 3 postoperative months, respectively. The mean preoperative ROM was 125.0° ± 9.1°, which improved to a mean of 126.6° ± 8.0° at final follow-up, although this difference was not statistically significant (*p* = 0.349). The mean HSS score increased from 59.5 ± 8.9 to 88.3 ± 9.3 at the time of final follow-up (*p* = 0.000). Postoperative HSS scores were significantly higher in group B (92.4 ± 4.5 compared with 84.3 ± 11.1 in group A, *p* = 0.002; Table 2).

**Table 2:** **UKA Demographics**

	**A (** ***N*** **= 25)**	**B (** ***N*** **= 25)**	***p*** **value**
Age (years)	62.8 ± 9.4	66.1 ± 7.2	0.168
Sex (male/female)	9:16	7:18	0.544
Side (right/left)	12:13	15:10	0.395
BMI (kg/m^2^)	24.9 ± 1.3	23.8 ± 4.1	0.316
Duration of surgery (min)	85.0 ± 15.7	64.4 ± 13.9	0.000
Length of incision (cm)	9.7 ± 1.3	8.5 ± 1.1	0.001
Blood loss (ml)	226.2 ± 74.8	185.0 ± 69.2	0.049
Preoperative HSS score	57.8 ± 8.5	61.2 ± 9.1	0.189
Preoperative range of motion	126.3 ± 7.6	123.7 ± 10.3	0.306
Postoperative HSS score	84.3 ± 11.1	92.4 ± 4.5	0.002
Postoperative range of motion	125.0 ± 9.0	128.2 ± 6.7	0.160

**Figure 1:**
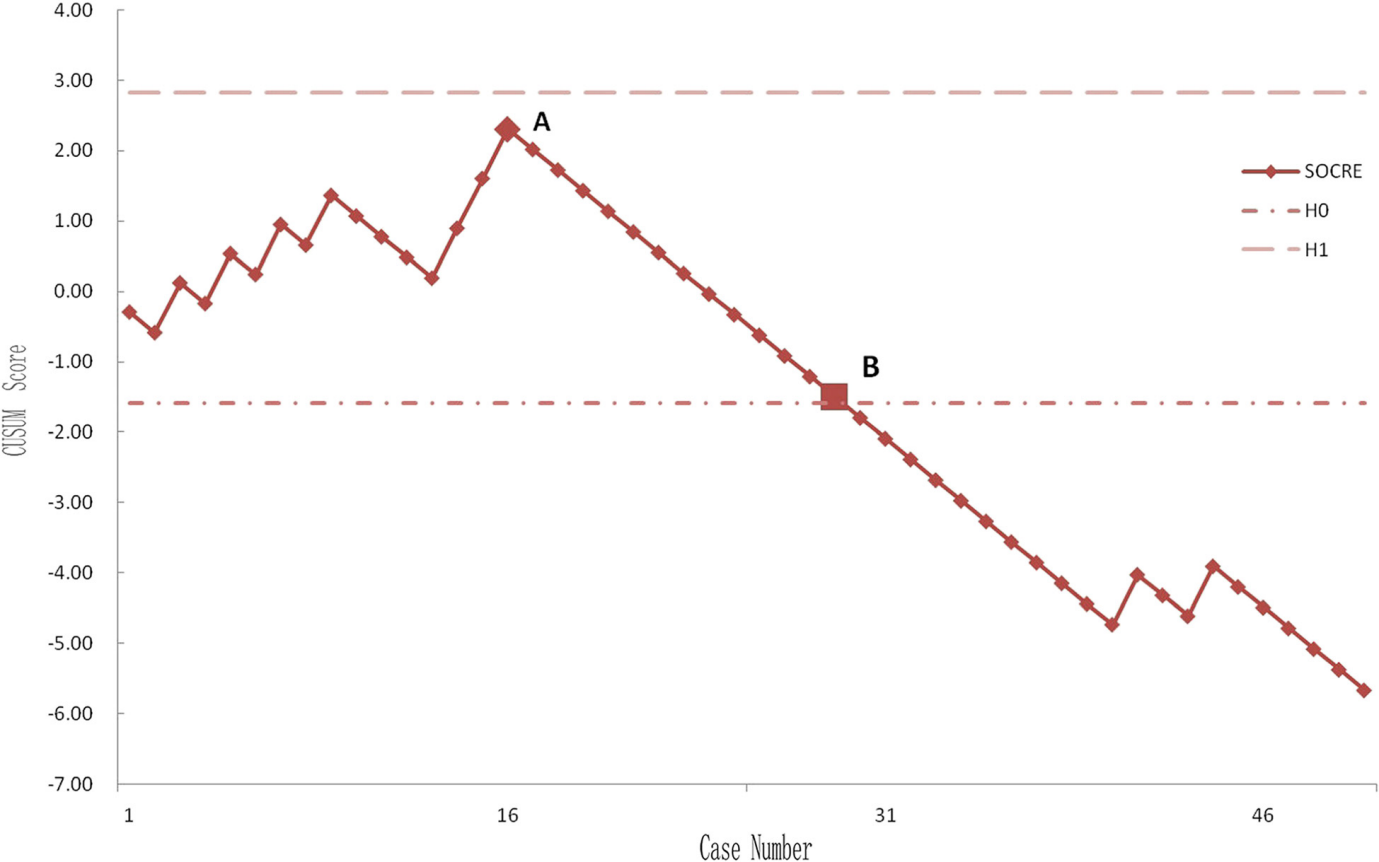
**CUSUM learning curve.** Point A (case 16) corresponds to the main inflection point at which the failure rate begins to keep consistent. At point B (case 29), the line crosses the lower decision limit and the failure rate is equal to the defined acceptable failure rate (20%). The failure rate did not reach the unacceptable threshold (h1) at any time.

**Figure 2:**
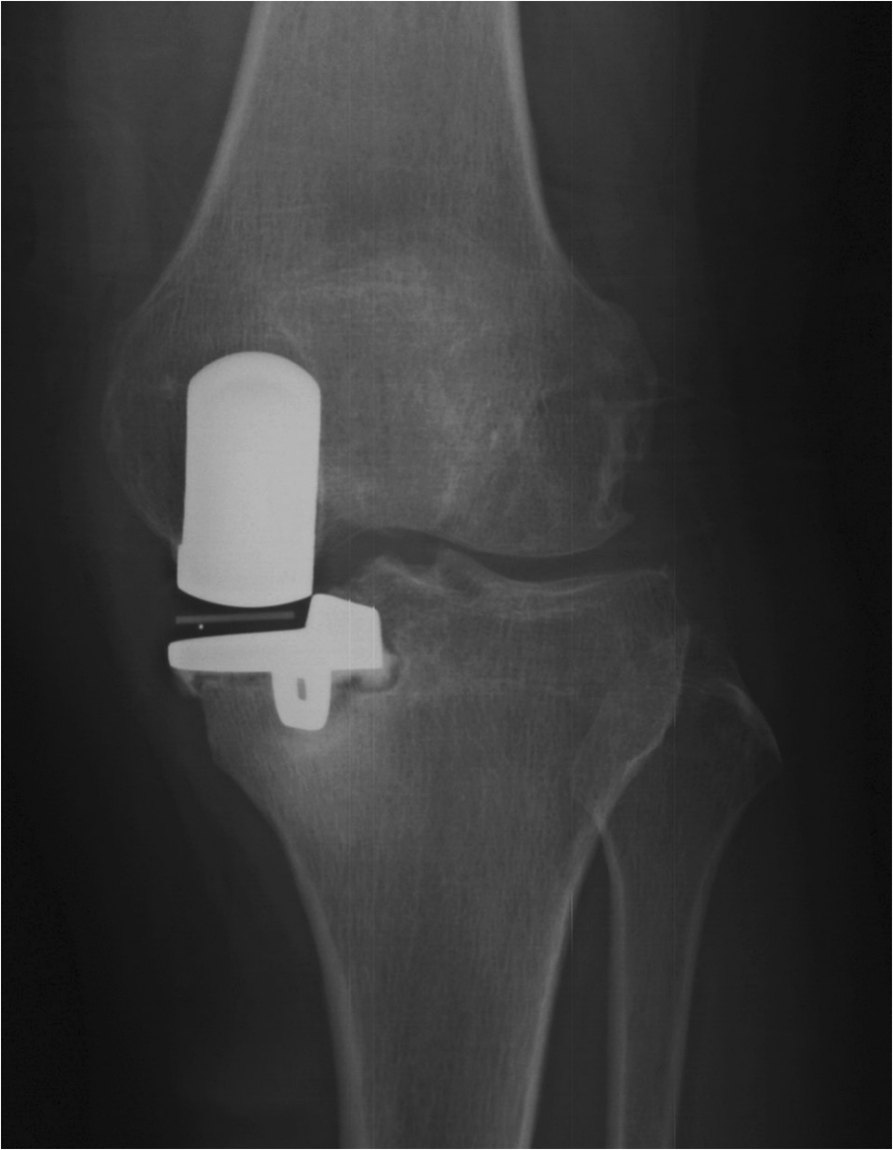
**Anteroposterior X-ray of postoperative UKA infection.**

**Figure 3:**
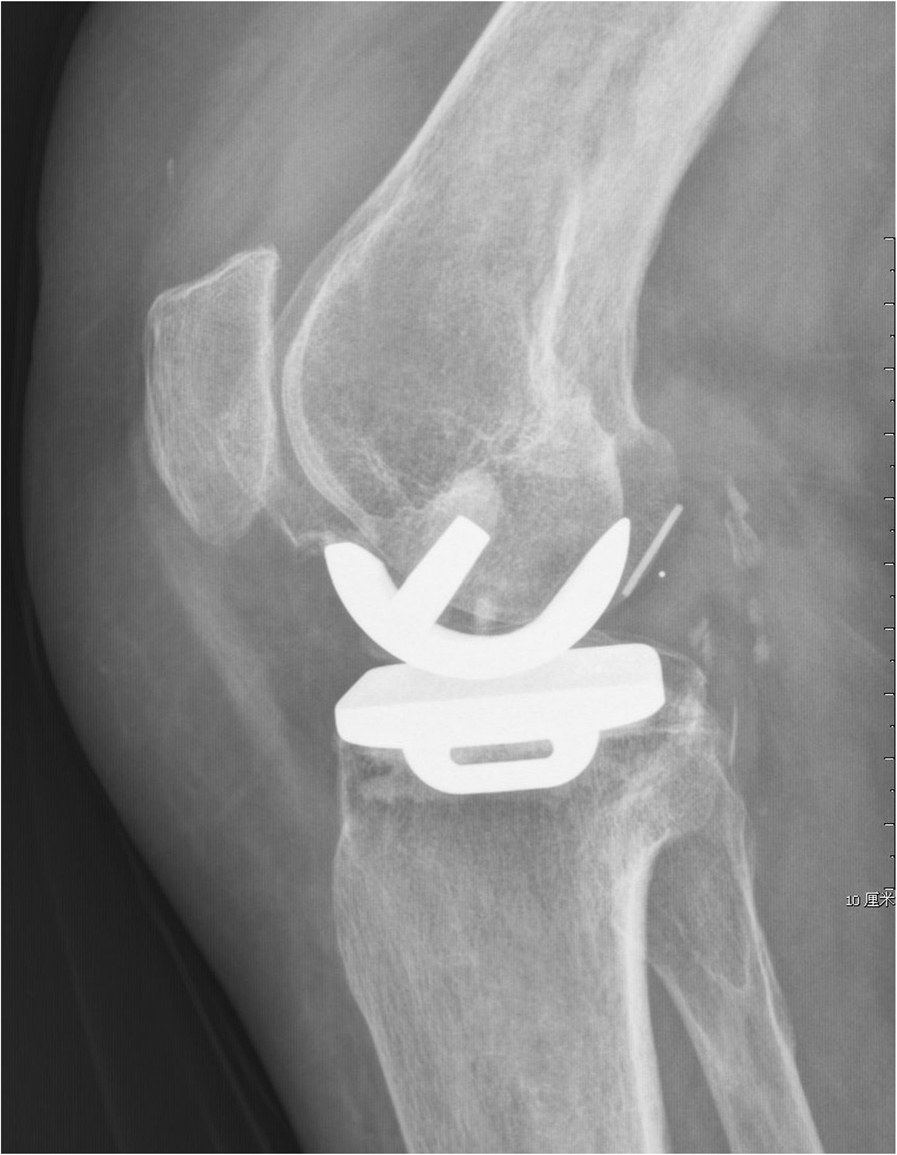
**Posterior dislocation of the bearing.**

**Figure 4:**
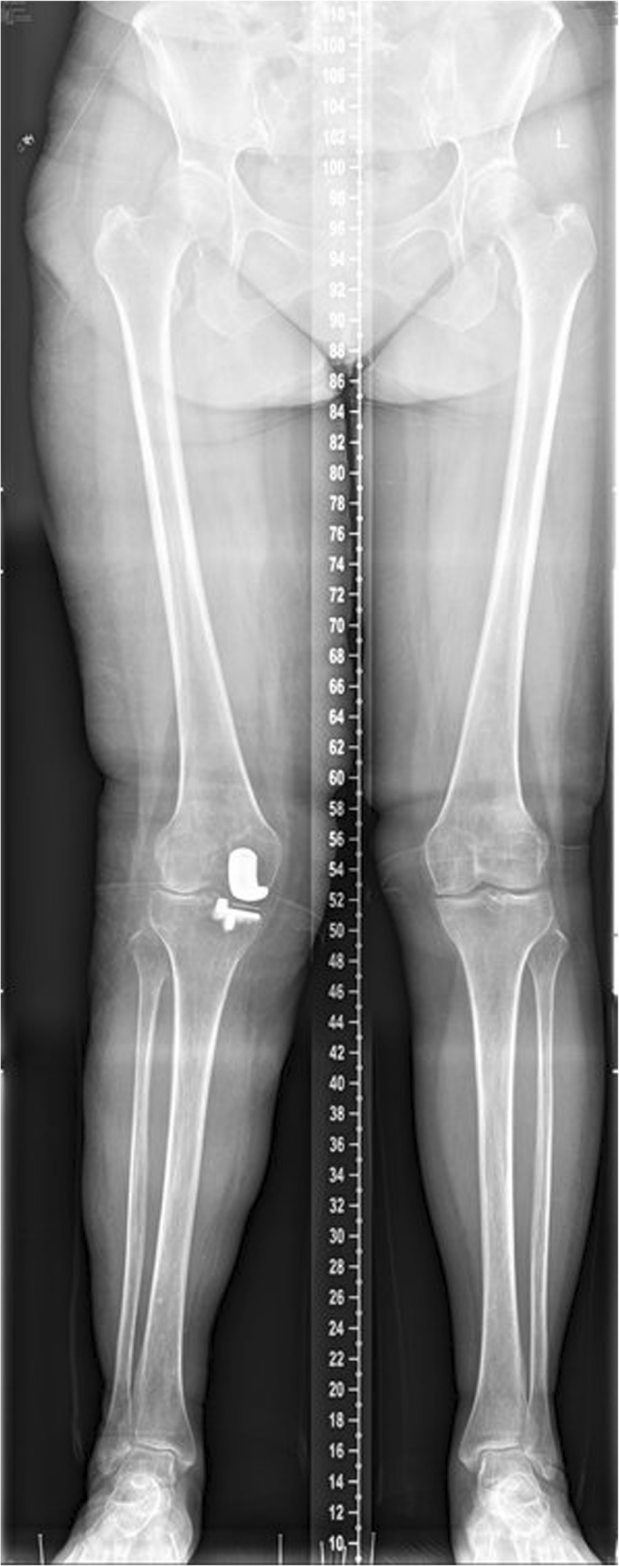
**Lateral compartment degeneration with valgus malalignment status after UKA.**

The CUSUM learning curve is shown in Figure 1 and Table 3. Failures were identified in nine patients (18%). There was a trend toward a higher failure rate during the first 25 cases, although this did not achieve statistical significance (28% in group A compared with 8% in group B; *p* = 0.138). Three of the 50 prostheses (6%) included in the analysis had been revised before final follow-up; with the end point of revision for any reason, the survival rate was 94%. In group A, one UKA was revised to a TKA as a consequence of aseptic loosening of the tibial component 3.5 years after surgery and one was revised to a TKA due to infection 1 year after surgery. In group B, one UKA was revised to a TKA after 4 years as a result of a lateral tibial plateau fracture sustained in a major trauma. There had been no clinical symptoms of implant failure or radiographic signs of loosening before the accident. Bearing dislocation occurred in two cases 1.5 and 2 years after surgery because of laxity after hyperflexion trauma; the bearings were replaced by thicker ones. No recurrences of luxation were seen at follow-up. One diagnosis of lateral compartment osteoarthritis was made in group A (Figures 2, 3, 4). Two patients in each group reported continuing unexplained pains. There were no serious adverse events, such as pulmonary embolism, deep venous thrombosis, or iatrogenic neurovascular injury. According to the guidelines proposed by the Oxford group [14], postoperative radiographic assessments showed that one of the components in group A was not in an acceptable position.

**Table 3:** **CUSUM chart calculations**

**Case**	**Sex**	**Age (year)**	**CUSUM score**	**Failure model**
1	M	60	−0.29	
2	F	57	−0.59	
3	F	55	0.12	Unexplained pain
4	M	76	−0.17	
5	F	49	0.53	Revision for infection
6	M	52	0.24	
7	F	50	0.95	Revision for aseptic loosening
8	F	67	0.65	
9	F	71	1.36	Malposition
10	F	52	1.07	
11	M	75	0.77	
12	F	56	0.48	
13	F	54	0.19	
14	F	61	0.89	Bearing dislocation
15	F	61	1.60	Lateral compartment degeneration
16	F	54	2.31	Bearing dislocation
17	M	74	2.01	
18	M	70	1.72	
19	F	72	1.43	
20	M	73	1.13	
21	F	69	0.84	
22	M	70	0.55	
23	F	70	0.25	
24	M	57	−0.04	
25	F	79	−0.33	
26	F	65	−0.63	
27	F	75	−0.92	
28	F	72	−1.21	
29	M	61	−1.51	
30	M	59	−1.80	
31	F	61	−2.09	
32	M	64	−2.39	
33	M	64	−2.68	
34	F	57	−2.97	
35	F	76	−3.27	
36	F	82	−3.56	
37	F	67	−3.85	
38	F	62	−4.15	
39	F	71	−4.44	
40	F	56	−4.73	
41	M	57	−4.03	Fracture
42	F	70	−4.32	
43	M	62	−4.61	
44	F	55	−3.91	Unexplained pain
45	F	78	−4.20	
46	F	58	−4.49	
47	F	73	−4.79	
48	F	61	−5.08	
49	F	70	−5.37	
50	M	61	−5.67

The CUSUM learning curve chart (Figure 1) shows that case 16 corresponds to the main inflection point (point A) at which the failure rate became consistent. At point B (case 29), the line crosses the lower decision limit and the failure rate is equal to the defined acceptable failure rate (20%). The failure rate did not reach the unacceptable threshold (h1) at any time.

## Discussion

Unicompartmental arthroplasty is a well-recognized treatment option for unicompartmental osteoarthritis of the knee. The Oxford unicompartmental knee has a mobile bearing with full congruency, minimizing polyethylene wear, and there are many reports of excellent postoperative outcomes [2,3,15-17]. Svard and Price reported a 95% cumulative survival rate over 10 years [4]. Pandit et al. reported the outcomes of 1,000 phase 3 Oxford medial UKAs using a minimally invasive surgical approach by two surgeons. Using revision as the end point, the 10-year survival rate was 99.8% [18]. In 2011, Price et al. reported the second decade data of the Oxford UKA, having previously reported longitudinal data from 1, 6, and 10 postoperative years [15,19,20]. In the most recent study, postoperative function and HSS score had still significantly improved from baseline. These findings suggest that the Oxford UKA is a reliable treatment option for anteromedial osteoarthritis of the knee.

Despite these apparent advantages, other investigators have reported less favorable long-term results or early failures that have required revision or reoperation [21-24]. The minimally invasive medial Oxford UKA is undoubtedly a demanding procedure, and many early failures are often due to technical errors [25]. Kort and colleagues [5] assessed 130 patients with minimally invasive Oxford phase 3 UKAs over a follow-up period of 2–7 years and reported an overall survival rate of 89%. Of 17 failures, they attributed 13 to human error, confirming that surgical expertise influences mid-term results and thereby long-term outcomes. Similarly, Kuipers and colleagues [26] reported that the cumulative survival rate of a cohort of 437 Oxford phase 3 implants was 84.7% at 5 years and that 101 of these were at risk. Each surgeon in the study performed an average of eight UKAs per year. In 2001, Robertson reported that centers that performed fewer than 23 UKAs annually had a revision risk 1.63 times higher than centers with higher volumes of casework [27]. Surgical experience therefore appears to be a critical determinant of outcome. These results also underline the existence of a learning curve for UKA and that minimally invasive Oxford phase 3 UKA is a demanding procedure that nonetheless has the potential to achieve satisfying surgical outcomes.

In our study, the five patients who required reoperations were all young: their ages at operation were 49, 50, 54, 57, and 61 years. Young patients are more likely to be physically active after surgery, which may be a risk factor for failure [26,28]. The patient in group B who was not satisfied with her outcome was of perimenopausal age, and it is possible that patients with endocrine-sensitive disease might not achieve such favorable surgical outcomes. It is very important that patients have realistic expectations about the extent of pain relief, the amount of physical activity permitted after surgery, and the likely duration of recovery following UKA in order to achieve high satisfaction rates.

As with the slope of the learning curve, the duration of surgery, blood loss, and length of incision gradually improved with the cumulative number of procedures. Nonetheless, we experienced many challenges due to the complexity of surgery. In summary, we found that it is essential (but may be difficult) to equalize the 90° and 20° flexion gaps. The principles of correct alignment and joint stability that are so important for TKA are likely to be at the forefront of the surgeon’s mind. To achieve stability, we elected to create a small tight extension gap in the early cases, which likely accounts for the one case of overcorrection in group A. Overcorrection of the tibiofemoral deformity can lead to failure because of degenerative change in the contralateral compartment [29]. Concern about this potential complication meant that we changed our practice so that a small but loose extension gap was acceptable. Undercorrection with a smaller bearing can cause bearing dislocation and increase the load to the medial compartment, which may accelerate polyethylene wear [25]. In our early cases, two bearing dislocations were encountered because of laxity, which might have been due to incomplete gap balancing between flexion and extension. In our cases of bearing dislocation, the bearings were replaced by new thicker ones. Although no recurrences of luxation occurred, osteoarthritis may progress in the lateral compartment in the future due to overcorrection of the alignment. A longer-term study is needed to examine this issue.

**Figure 5:**
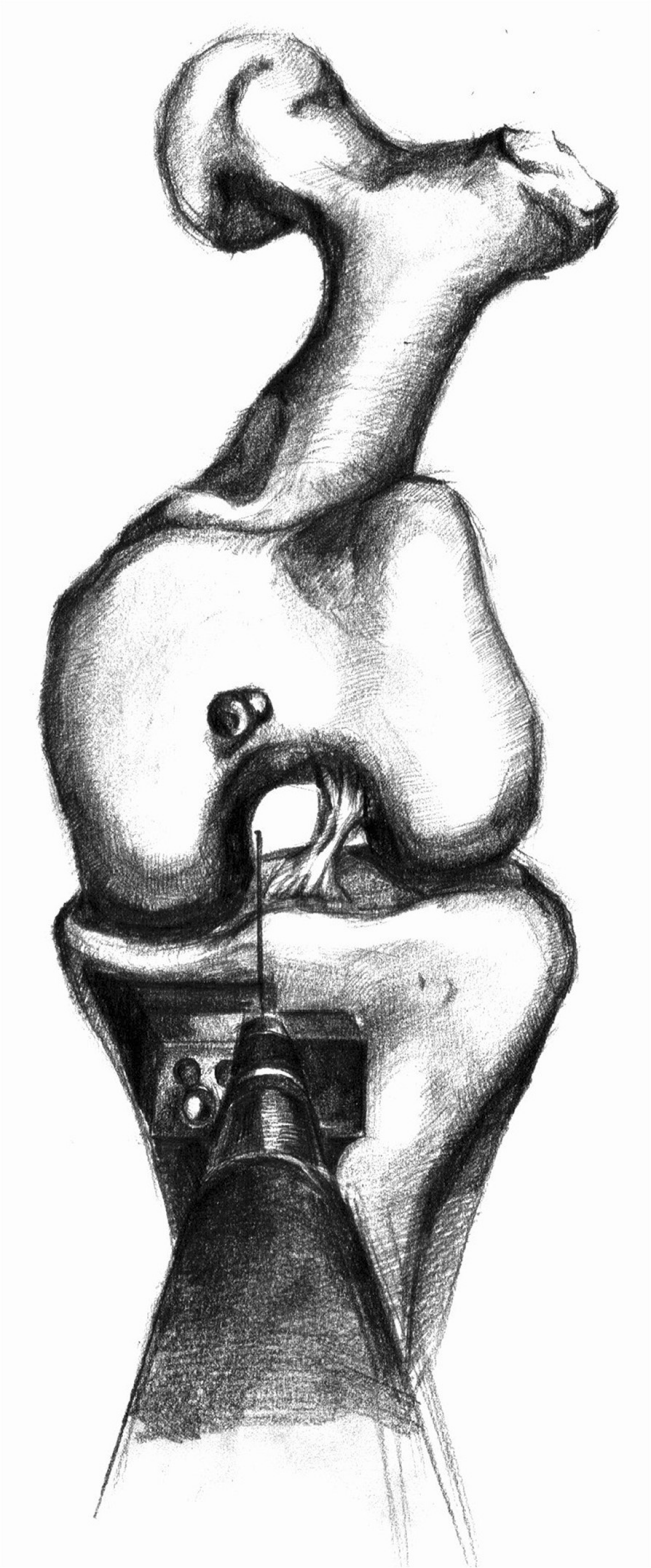
**The intramedullary rod was used for a guide.**

**Figure 6:**
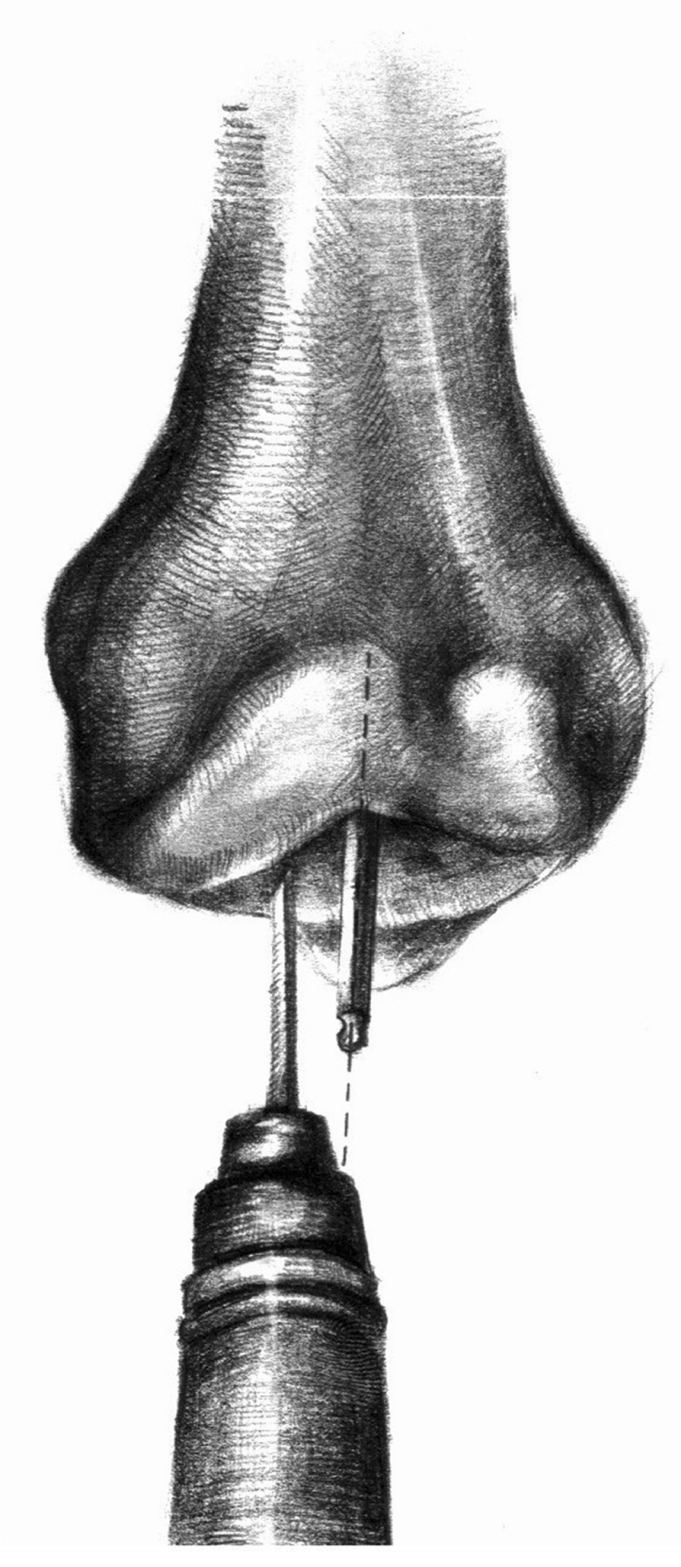
**The axis of blade lies 7° medial to the intramedullary rod when viewed from above.**

One essential surgical objective is accurate positioning of the components according to the guidelines proposed by the Oxford group [14]. There are many factors that must be assessed on the postoperative radiographs. The tibial component should be positioned just medial to the apex of the medial spine and should extend to the medial margin of the tibia or overhang by up to 2 mm. It should also reach the posterior cortex of the tibia. The bearing should be a few millimeters away from the vertical wall. Malalignment of the femoral component of up to 10° and of the tibial component of up to 5° is considered acceptable. In the earlier cases, we found the vertical bone cut in the tibial plateau challenging. The Oxford group’s guidelines state that the blade should point to the head of the femur; however, we found it difficult to locate the position of the head of the femur accurately. Consequently, postoperative radiographs taken to assess implant positioning showed that one implant was not within the recommended limits for positioning criteria. We modified our technique and used the intramedullary rod as a guide. The axis of the blade lay 7° medial to the intramedullary rod when viewed from above (Figures 5 and 6). With this technique, the number of malposition was reduced.

Our study has some limitations. First, by virtue of this type of study, the follow-up time of group B was shorter than that of group A, which may have influenced our ability to detect some longer-term complications. Second, we examined the outcomes of a single experienced knee surgeon, so our findings may not be representative of surgeons with other levels of expertise. Third, the follow-up time was relatively short, and a longer-term study is needed to confirm our results.

## Conclusions

Minimally invasive Oxford phase 3 UKA for medial unicompartmental knee osteoarthritis is a demanding procedure but only needs a short learning curve to achieve satisfactory surgical outcomes in the hands of an experienced knee surgeon. The mean duration of surgery, incision length, blood loss, and the incidence of postoperative complications decline with experience. Based on our CUSUM analysis, 25 cases are required before a consistently low failure rate is achieved for minimally invasive Oxford phase 3 UKA.

## Abbreviations

UKA: unicompartmental knee arthroplasty
LC-CUSUM: cumulative summation test for learning curve
TKA: total knee arthroplasty
HSS: Hospital for special surgery
ACL: anterior cruciate ligament

## References

[1] Berger RA, Della VC (2010). Unicompartmental knee arthroplasty: indications, techniques, and results. Instr Course Lect.

[2] Lisowski LA, van den Bekerom MP, Pilot P, van Dijk CN, Lisowski AE (2011). Oxford Phase 3 unicompartmental knee arthroplasty: medium-term results of a minimally invasive surgical procedure. Knee Surg Sports Traumatol Arthrosc.

[3] Price AJ, Dodd CA, Svard UG, Murray DW (2005). Oxford medial unicompartmental knee arthroplasty in patients younger and older than 60 years of age. J Bone Joint Surg (Br).

[4] Svard UC, Price AJ (2001). Oxford medial unicompartmental knee arthroplasty. A survival analysis of an independent series. J Bone Joint Surg (Br).

[5] Kort NP, van Raay JJ, van Horn JJ (2007). The Oxford phase III unicompartmental knee replacement in patients less than 60 years of age. Knee Surg Sports Traumatol Arthrosc.

[6] Pandit H, Jenkins C, Barker K, Dodd CA, Murray DW (2006). The Oxford medial unicompartmental knee replacement using a minimally-invasive approach. J Bone Joint Surg (Br).

[7] Tang H, Zhao L, Yan H, Jin D, Su X (2012). Mid-term effectiveness of Oxford Unicompartmental Knee System Phase III for medial unicompartmental knee osteoarthritis. Zhongguo Xiu Fu Chong Jian Wai Ke Za Zhi.

[8] Dionigi G, Bacuzzi A, Boni L, Rovera F, Dionigi R (2008). What is the learning curve for intraoperative neuromonitoring in thyroid surgery?. Int J Surg.

[9] Walton R, Theodorides A, Molloy A, Melling D (2012). Is there a learning curve in foot and ankle surgery?. Foot Ankle Surg.

[10] Murray DW (2005). Mobile bearing unicompartmental knee replacement. Orthopedics.

[11] Nayak BK, Hazra A (2011). How to choose the right statistical test?. Indian J Ophthalmol.

[12] Biau DJ, Porcher R (2010). A method for monitoring a process from an out of control to an in control state: application to the learning curve. Stat Med.

[13] Lee YK, Ha YC, Hwang DS, Koo KH (2013). Learning curve of basic hip arthroscopy technique: CUSUM analysis. Knee Surg Sports Traumatol Arthrosc.

[14] Goodfellow JW, O’Connor J, Dodd CAF, Murray DW (2006). Unicompartmental arthroplasty with the Oxford knee.

[15] Price AJ, Svard U (2011). A second decade lifetable survival analysis of the Oxford unicompartmental knee arthroplasty. Clin Orthop Relat Res.

[16] Emerson RJ, Higgins LL (2008). Unicompartmental knee arthroplasty with the oxford prosthesis in patients with medial compartment arthritis. J Bone Joint Surg Am.

[17] Kim KT, Lee S, Park HS, Cho KH, Kim KS (2007). A prospective analysis of Oxford phase 3 unicompartmental knee arthroplasty. Orthopedics.

[18] Pandit H, Jenkins C, Gill HS, Barker K, Dodd CA, Murray DW (2011). Minimally invasive Oxford phase 3 unicompartmental knee replacement: results of 1000 cases. J Bone Joint Surg (Br).

[19] Price AJ, Short A, Kellett C, Beard D, Gill H, Pandit H, Dodd CA, Murray DW (2005). Ten-year in vivo wear measurement of a fully congruent mobile bearing unicompartmental knee arthroplasty. J Bone Joint Surg (Br).

[20] Price AJ, Waite JC, Svard U (2005). Long-term clinical results of the medial Oxford unicompartmental knee arthroplasty. Clin Orthop Relat Res.

[21] Edmondson MC, Isaac D, Wijeratna M, Brink S, Gibb P, Skinner P (2011). Oxford unicompartmental knee arthroplasty: medial pain and functional outcome in the medium term. J Orthop Surg Res.

[22] Munk S, Odgaard A, Madsen F, Dalsgaard J, Jorn LP, Langhoff O, Jepsen CF, Hansen TB (2011). Preoperative lateral subluxation of the patella is a predictor of poor early outcome of Oxford phase-III medial unicompartmental knee arthroplasty. Acta Orthop.

[23] Rea P, Short A, Pandit H, Price AJ, Kyberd P, Beard DJ, Gill HS, Murray DW (2007). Radiolucency and migration after Oxford unicompartmental knee arthroplasty. Orthopedics.

[24] Saldanha KA, Keys GW, Svard UC, White SH, Rao C (2007). Revision of Oxford medial unicompartmental knee arthroplasty to total knee arthroplasty - results of a multicentre study. Knee.

[25] Lewold S, Goodman S, Knutson K, Robertsson O, Lidgren L (1995). Oxford meniscal bearing knee versus the Marmor knee in unicompartmental arthroplasty for arthrosis. A Swedish multicenter survival study. J Arthroplasty.

[26] Kuipers BM, Kollen BJ, Bots PC, Burger BJ, van Raay JJ, Tulp NJ, Verheyen CC (2010). Factors associated with reduced early survival in the Oxford phase III medial unicompartment knee replacement. Knee.

[27] Robertsson O, Knutson K, Lewold S, Lidgren L (2001). The routine of surgical management reduces failure after unicompartmental knee arthroplasty. J Bone Joint Surg (Br).

[28] W-Dahl A, Robertsson O, Lidgren L, Miller L, Davidson D, Graves S (2010). Unicompartmental knee arthroplasty in patients aged less than 65. Acta Orthop.

[29] Padgett DE, Stern SH, Insall JN (1991). Revision total knee arthroplasty for failed unicompartmental replacement. J Bone Joint Surg Am.

